# Guidelines for reporting embedded recruitment trials

**DOI:** 10.1186/s13063-015-1126-y

**Published:** 2016-01-14

**Authors:** Vichithranie W. Madurasinghe, Sandra Eldridge on behalf of MRC START Group and Gordon Forbes on behalf of the START Expert Consensus Group

**Affiliations:** Pragmatic Clinical Trials Unit (PCTU), Centre for Primary Care and Public Health, Blizard Institute, Yvonne Carter Building, 58 Turner Street, London, E1 2AB UK

**Keywords:** Embedded randomised controlled trial, Recruitment, Primary care, Reporting guidelines, Methodology

## Abstract

**Background:**

Recruitment to clinical trials is difficult with many trials failing to recruit to target and within time. Embedding trials of recruitment interventions within host trials may provide a successful way to improve this. There are no guidelines for reporting such embedded methodology trials. As part of the Medical Research Council funded Systematic Techniques for Assisting Recruitment to Trials (MRC START) programme designed to test interventions to improve recruitment to trials, we developed guidelines for reporting embedded trials.

**Methods:**

We followed a three-phase guideline development process: (1) pre-meeting literature review to generate items for the reporting guidelines; (2) face-to-face consensus meetings to draft the reporting guidelines; and (3) post-meeting feedback review, and pilot testing, followed by finalisation of the reporting guidelines.

**Results:**

We developed a reporting checklist based on the Consolidated Standards for Reporting Trials (CONSORT) statement 2010. Embedded trials evaluating recruitment interventions should follow the CONSORT statement 2010 and report all items listed as essential. We used a number of examples to illustrate key issues that arise in embedded trials and how best to report them, including (a) how to deal with description of the host trial; (b) the importance of describing items that may differ in the host and embedded trials (such as the setting and the eligible population); and (c) the importance of identifying clearly the point at which the recruitment interventions were embedded in the host trial.

**Conclusions:**

Implementation of these guidelines will improve the quality of reports of embedded recruitment trials while advancing the science, design and conduct of embedded trials as a whole.

**Electronic supplementary material:**

The online version of this article (doi:10.1186/s13063-015-1126-y) contains supplementary material, which is available to authorized users.

## Background

Randomised controlled trials (RCTs) are considered as the ‘gold standard’ for evaluating health technologies, but recruitment to trials remains problematic with the majority failing to recruit to target and within time [1, 2]. This results in either underpowered trials that cannot produce robust results or, in some cases, extended recruitment, which adds additional expense.

In spite of this there are very few trials evaluating different strategies for recruiting clinicians and patients to clinical trials. Recent systematic reviews of trials assessing interventions for improving recruitment to trials found only a limited numbers of relevant studies [3–5]. Furthermore, many of the included trials were small, likely to be underpowered and unable to determine with any certainty the magnitude of benefit. This is particularly true for interventions that modified the method for approaching potential participants. In addition, some of the included studies were hypothetical trials where the host trial that eligible patients were recruited to did not exist, and the implications of their results for real trials are unclear [3].

Based on their findings, the review authors argued that while the use of hypothetical trials to study recruitment interventions has its place, trialists should include evaluations of recruitment strategies within their trials, and research funding bodies should support this as part of future trial methodologies [3]. The lack of definitive results to make strong recommendations was also highlighted in a Health Technology Assessment (HTA) into recruitment to clinical trials [6]. The report recommended that future research should focus on formally evaluating strategies aimed at increasing recruitment and making trials more successful for their effectiveness in a range of trials.

One way of doing this is to conduct trials of recruitment strategies within host trials; that is to conduct embedded recruitment trials. In order to maximise the impact of embedded recruitment trials to the research community, they need to be well reported. Critical appraisal of the quality of clinical trials is possible only if the design, conduct, and analysis of RCTs are thoroughly and accurately described in published articles. Far from being transparent, the reporting of RCTs is often incomplete [7]. Biased results from poorly designed and reported trials can mislead decision-making in health care at all levels, from treatment decisions for the individual patient to formulation of national public health policies [7].

A systematic review by Caldwell et al. [8] found that, of the 37 embedded recruitment trials included, only 12 trials (32 %) clearly reported allocation concealment, two (4 %) specified blinding of outcome assessors (no trial had blinding of participants to intervention received as this would have been difficult to achieve), 15 (40 %) recorded loss to follow-up information, and 14 (38 %) used intention-to-treat analysis. Similar findings were reported by Treweek et al. [3], who found that although all trials in their review were described by their authors as being either randomised (*n* = 41) or quasi-randomised (*n* = 4), more than a third failed to provide details of the method used to achieve this. Similarly, while allocation concealment was adequate in half of the trials, details were poorly reported in many others. This was also true in relation to the procedures used to blind participants, which was often missing or not fully reported. When considered across the domains, 12 trials had a low risk of bias, 13 had moderate risk and 20 had a high risk.

In addition to being poorly reported, embedded trials have some methodological characteristics that are atypical when compared with trials of effectiveness (such as eligible population, sample size). These characteristics affect their design, conduct, interpretation and reporting. Currently there are no guidelines for reporting embedded trials.

The Medical Research Council funded Systematic Techniques for Assisting Recruitment to Trials (MRC START) programme is designed to develop the conceptual and logistical framework for conducting embedded recruitment trials and to assess their feasibility. It aims to improve the evidence base concerning recruitment to trials by developing a small number of recruitment interventions and testing these across multiple host RCTs, enhance recruitment rates and make research more accessible to the public [9]. As part of the MRC START programme we aim to develop guidelines for reporting embedded recruitment trials. This manuscript describes these guidelines and how they were developed.

We defined an embedded recruitment trial as a RCT in which an intervention (or several interventions) to enhance recruitment outcomes are tested in the context of another RCT (or several RCTs) known as the host RCT(s). Design and conduct of the embedded trial is often constrained by its host RCT(s). It is important to note that embedded recruitment trials may include retention as an outcome; however, trials where embedded intervention is aiming to improve retention exclusively are excluded.

## Methods

These guidelines were developed through an iterative process following the general recommendations of the EQUATOR (Enhancing the Quality and Transparency of health Research) network on how to develop a reporting guideline [10]. Systematic reviews by members of the MRC START team [3] and others [8] had already highlighted the need for these guidelines.

### Generating items for inclusion in the checklist

Initial criteria were drawn up by SE, from reading nine trial reports. The trials selected purposively were (a) one very recent embedded recruitment trial with an MRC START investigator as co-applicant [11] and thus likely to provide a good indicator of the most up-to-date reporting practice; (b) three recent trials of multi-media interventions likely to be common in the future, and (c) five recent embedded trials from a large systematic review deemed to be at low risk of bias (and therefore we assumed high quality). A second researcher (VM) compared the initial criteria against the standard Consolidated Standards for Reporting Trials (CONSORT) statement 2010 [12] and identified items that could be matched directly to the CONSORT checklist. Criteria that could not be matched were identified as potential new items. These items were then mapped to a CONSORT 2010 section heading.

These initial criteria were then presented to the MRC START programme team at a face-to-face meeting for discussion. The team suggested that these reporting guidelines should extend to cover cluster randomised embedded trials; nearly half of the embedded recruitment trials in MRC START programme used cluster randomised design (e.g. randomised to recruitment intervention at practice or site level). We also agreed that these guidelines should focus on prompting authors to provide as much information as possible for readers, to facilitate the adaption of successful recruitment strategies in future trials. Therefore, the MRC START team agreed to add the proposed new items to the provisional checklist. It was agreed that, although these guidelines could apply to other embedded trials, the primary focus of this work should be on embedded *recruitment* trials.

Following these discussions, the initial checklist was revised to incorporate cluster randomised embedded trials, two new items were added ((1) a brief description of host trial and (2) methods of collecting data) and, where appropriate, item descriptors were amended so that they were applicable to embedded trials. The revised checklist contained 40 items. Where possible, good examples of embedded trial reporting were added under each item.

### Consensus meetings and pilot testing

We organised two face-to-face consensus meetings. The first one was held in Manchester, UK in August 2014. This meeting was open to the MRC START research team and chief investigators of host trials involved in evaluating MRC START recruitment interventions. Seven people agreed to participate. Paper copies of the revised checklist with examples were circulated to attendees.

SE and VM introduced each item to the group and presented the rationale for inclusion, encouraging discussion. During the meeting, discussions of each potential item continued. As this work is partly designed to support trial teams in writing up MRC START embedded trials for publication, participants were encouraged to share their views on how each item might apply to their own trial and any reservations they had, usefulness and any difficulties that they could foresee in applying these guidelines to their own trials.

By the end of the discussions, we sought to come to an agreement on each item in terms of whether it should be included as a checklist item, how each item may apply to embedded trials, justification for inclusion and descriptor wording. At the end of the meeting, two trial teams agreed to pilot-test the preliminary reporting guidelines. Before the second consensus meeting, draft reporting guidelines were updated incorporating the feedback from the day and from the pilot trials.

The second consensus meeting was held in Manchester, UK in October 2014. The purpose of this meeting was to finalise the provisional reporting checklist. External experts as well as members of the MRC START Group were invited to this meeting. Attendees included review authors, methodologists, statisticians, medical journal editors and chief investigators or managers from MRC START host trials. The revised draft reporting guidelines document was circulated to all who agreed to attend before the meeting. Thirteen people attended. Following preliminary introductions to the MRC START programme and its objectives, SE introduced each checklist item, presented the rationale for inclusion and opened the floor for discussion. During the meeting, arguments for and against the inclusion of each potential item continued. At the meeting, we sought to reach consensus on the final checklist, descriptor wording and explanations. It was agreed that this guideline should be used in conjunction with other relevant reporting guidelines, in line with CONSORT recommendations [7]. Thus, specific additions relating to cluster randomised trials were removed.

### Post-meeting activities

After the meeting, SE and VM met on three occasions to finalise the descriptor wording, explanations and examples. Revised reporting guidelines were circulated via e-mail to all attendees (consensus meeting 1 and 2 attendees) for comments. All suggestions for revision were addressed.

Further, we reviewed the CONSORT guidelines for abstracts against the finalised checklist items for reporting embedded recruitment trials to identify any additional items that need to be reported within an abstract of an embedded trial as essential.

### Recommendations for reporting embedded recruitment trials

The revised checklist for reporting embedded recruitment trials was developed in line with the CONSORT statement 2010 [12]. The CONSORT statement provides a list of essential items to include when reporting randomised trials. Our revised checklist does not include additional items to be reported as essential for embedded recruitment trials. However, we have extended some items to cover specific reporting requirements of these trials (Table 1). Further, we have provided extensive explanations for such recommendations made. We have used a number of examples to illustrate how embedded trials may adhere to those CONSORT items particularly relevant to them and specific requirements that we have highlighted. These include how best to deal with the description of the host trial, the importance of describing items that may differ in the host and embedded trials such as the setting and the eligible population, and the importance of identifying clearly the point at which the recruitment intervention embedded in the host trial. Further examples of good reporting are provided in Additional file 1.

**Table 1:** Checklist of items for reporting embedded recruitment trials

Section/topic and item no.	CONSORT 2010 (standard) checklist item	Extension for embedded recruitment trials
Title and abstract		
1a	Identification as a randomised trial in the title	Identification as an *embedded randomised recruitment trial* in the title
1b	Structured summary of trial design, methods, results, and conclusions (for specific guidance see CONSORT for abstracts)	Structured summary of *embedded recruitment trial* design, methods, results, and conclusions (for specific guidance see CONSORT for abstracts)
Introduction		
Background and objectives		
2a	Scientific background and explanation of rationale	Scientific background and explanation of rationale for the *embedded recruitment trial including a brief description of the host trial(s) as appropriate*
2b	Specific objectives or hypotheses	Specific objectives or hypotheses for the *embedded recruitment trial*
Methods		
Trial design		
3a	Description of trial design (such as parallel, factorial) including allocation ratio	Description of *embedded recruitment trial* design (such as parallel, factorial, *cluster*) including allocation ratio
3b	Important changes to methods after trial commencement (such as eligibility criteria), with reasons	Important changes to methods of the *embedded recruitment trial* after commencement (such as eligibility criteria), with reasons
Participants		
4a	Eligibility criteria for participants	Eligibility criteria for participants for the *embedded recruitment trial, including any differences from those for the host trial(s)*
4b	Settings and locations where the data were collected	Settings and locations where the *embedded recruitment trial was carried out, including a brief description of the host trial(s) as appropriate*
Interventions		
5	The interventions for each group with sufficient details to allow replication, including how and when they were actually administered	The interventions for each group *(including control group) within the embedded recruitment trial* with sufficient details to allow replication, including how, where and when they were actually administered
Outcomes		
6a	Completely defined pre-specified primary and secondary outcome measures, including how and when they were assessed	Completely defined pre-specified primary and secondary outcome measures for the *embedded recruitment trial,* including how and when they were assessed
6b	Any changes to trial outcomes after the trial commenced, with reasons	Any changes to *embedded recruitment* trial outcomes after the *embedded recruitment trial* commenced, with reasons
Sample size		
7a	How sample size was determined	How sample size for the *embedded recruitment trial* was determined
7b	When applicable, explanation of any interim analyses and stopping guidelines	When applicable, explanation of any interim analyses and stopping guidelines for the *embedded recruitment trial*
Randomisation		
Sequence generation		
8a	Method used to generate the random allocation sequence	Method used to generate the random allocation sequence for the *embedded recruitment trial*
8b	Type of randomisation; details of any restriction (such as blocking and block size)	Type of randomisation; details of any restriction (such as blocking and block size) in the *embedded recruitment trial*
Allocation concealment mechanism		
9	Mechanism used to implement the random allocation sequence (such as sequentially numbered containers), describing any steps taken to conceal the sequence until interventions were assigned	Mechanism used in the *embedded recruitment trial* to implement the random allocation sequence (such as sequentially numbered containers), describing any steps taken to conceal the sequence until interventions were assigned
Implementation		
10	Who generated the random allocation sequence, who enrolled participants, and who assigned participants to interventions?	Who generated the random allocation sequence(s), who enrolled participants, and who assigned participants to *embedded recruitment* interventions?
Blinding		
11a	If done, who was blinded after assignment to interventions (for example, participants, care providers, those assessing outcomes) and how?	If done, who was blinded after assignment to *embedded recruitment* interventions (for example, participants, care providers, those assessing outcomes) and how?
11b	If relevant, description of the similarity of interventions	If relevant, description of the similarity of interventions in the *embedded recruitment trial*
Statistical methods		
12a	Statistical methods used to compare groups for primary and secondary outcomes	Statistical methods used to compare groups for primary and secondary outcomes of the *embedded recruitment trial*
12b	Methods for additional analyses, such as subgroup analyses and adjusted analyses	Methods for additional analyses, such as subgroup analyses and adjusted analyses for the *embedded recruitment trial*
Results		
Participant flow (a diagram is strongly recommended)		
13a	For each group, the numbers of participants who were randomly assigned, received intended treatment, and were analysed for the primary outcome	For each group in the *embedded recruitment trial,* the numbers of participants who were randomly assigned, received intended treatment, and were analysed for the primary outcome
13b	For each group, losses and exclusions after randomisation, together with reasons	For each group, losses and exclusions after randomisation to the *embedded recruitment trial,* together with reasons
Recruitment		
14a	Dates defining the periods of recruitment and follow-up	Dates defining the periods of recruitment and follow-up *for both embedded recruitment trial and host trial(s)*
14b	Why the trial ended or was stopped	Why the *embedded recruitment trial* ended or was stopped
Baseline data		
15	A table showing baseline demographic and clinical characteristics for each group	*If possible* a table showing baseline characteristics *of each arm of the embedded recruitment trial*
Numbers analysed		
16	For each group, number of participants (denominator) included in each analysis and whether the analysis was by original assigned groups	For each group in the *embedded recruitment trial,* number of participants (denominator) included in each analysis and whether the analysis was by original assigned groups
Outcomes and estimation		
17a	For each primary and secondary outcome, results for each group, and the estimated effect size and its precision (such as 95 % confidence interval)	For each primary and secondary outcome, results for each group in the *embedded recruitment trial,* and the estimated effect size and its precision (such as 95 % confidence interval)
17b	For binary outcomes, presentation of both absolute and relative effect sizes is recommended	For binary outcomes in the *embedded recruitment trial*, presentation of both absolute and relative effect sizes is recommended
Ancillary analyses		
18	Results of any other analyses performed, including subgroup analyses and adjusted analyses, distinguishing pre-specified from exploratory	Results of any other analyses performed for the *embedded recruitment trial*, including subgroup analyses and adjusted analyses, distinguishing pre-specified from exploratory
Harms		
19	All important harms or unintended effects in each group (for specific guidance see CONSORT for harms)	All important harms or unintended effects in each group *for both the embedded recruitment trial and host trial(s)* (for specific guidance see CONSORT for harms)
Discussion		
Limitations		
20	Trial limitations, addressing sources of potential bias, imprecision, and, if relevant, multiplicity of analyses	*Embedded recruitment trial* limitations, addressing sources of potential bias, imprecision, and, if relevant, multiplicity of analyses
Generalisability		
21	Generalisability (external validity, applicability) of the trial findings	Generalisability (external validity, applicability) *of the embedded recruitment trial* findings
Interpretation		
22	Interpretation consistent with results, balancing benefits and harms, and considering other relevant evidence	Interpretation consistent with results *of the embedded recruitment trial,* balancing benefits and harms, and considering other relevant evidence
Other information		
Registration		
23	Registration number and name of trial registry	Registration number and name of trial registry *(for all host trials and embedded recruitment trial if available)*
Protocol		
24	Where the full trial protocol can be accessed, if available	Where the *embedded recruitment trial* protocol can be accessed, if available
Funding		
25	Sources of funding and other support (such as supply of drugs), role of funders	For the *embedded recruitment trial,* sources of funding and other support, role of funders *and collaborators*

Table 1 presents the checklist of items to include when reporting embedded recruitment trials. This article also provides the rationale and meaning of each criterion in the context of embedded recruitment trials and for most items, at least one published example of good reporting is provided. Several examples of CONSORT flow diagrams are also included. In these examples, authors’ references to other publications were removed to avoid confusion. The recommendations in this paper should be seen as additional to the general guidance in the main CONSORT explanatory paper [7] and, where relevant, other CONSORT guidance such as CONSORT extension to cluster randomised trials.

### Title and abstract

#### Item 1a

Standard CONSORT 2010 item: identification as a randomised trial in the title.

Extension for embedded recruitment trial: identification as an *embedded randomised recruitment trial* in the title.

Example:

Improving recruitment to a study of telehealth management for long-term conditions in primary care: two embedded RCTs of optimised patient information material [13].

Explanation:

The importance of RCTs that test the effectiveness of different approaches to recruitment has been highlighted: ‘The most robust test of the effectiveness of a recruitment method is an RCT comparing one method with an alternative, “nested” in a real “host” trial [9]’. Embedding RCTs within host RCTs not only provides a platform for conducting recruitment research but also could be used in other methodological research into design and conduct of RCTs [14], for example, research on retention in RCTs. Though embedded trials are less common than RCTs of treatment effectiveness, they provide much needed research evidence for trialists. We recognise that at present there are no widely used terms to distinguish these trials. Several reviews on trial methods have highlighted the challenges in identifying relevant research studies through electronic database searches [14, 15]. Consistent use of the term ‘embedded’ in trial titles would ensure that such methodological studies are more easily identifiable and distinguished from other RCTs.

We therefore strongly encourage adding the term ‘embedded randomised trial’ in the title to ensure that these trials are identifiable in electronic database searches. We prefer to use the term ‘embedded’ instead of ‘nested’, to ensure that these trials are not confused with other study designs such as nested case-control studies, where only a subgroup of the original study population is included in the nested study.

Further, we suggest that the term ‘recruitment’ is added to describe the focus of embedded interventions.

#### Item 1b

Standard CONSORT 2010 item: structured summary of trial design, methods, results, and conclusions (for specific guidance see CONSORT for abstracts [16]).

Extension for embedded recruitment trial: structured summary of *embedded recruitment trial* design, methods, results, and conclusions (for specific guidance see CONSORT for abstracts [16]).

**Fig. 1:**
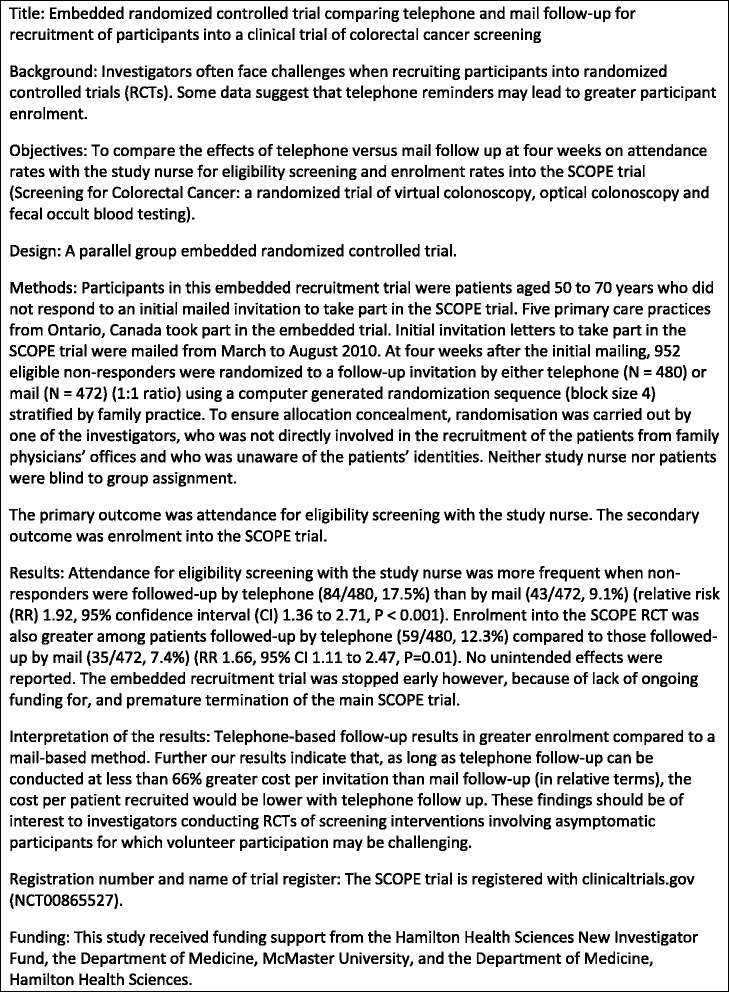
Example abstract for an embedded recruitment trial [17]

Example:

We recommend that embedded recruitment trials should follow the CONSORT abstract guidelines for reporting abstracts. We have used an abstract from a published journal article [17] to illustrate how embedded recruitment trials may strive to adhere to CONSORT abstract guidelines [16]. Here we have enhanced the original abstract to incorporate specific reporting requirements that we have recommended for embedded recruitment trials (see Fig. 1).

Explanation:

No additional items were identified for reporting within the abstracts of embedded recruitment trials. However, authors must pay special attention to specific requirements that we have highlighted for some of the essential items in the checklist. Additional information that we have recommended on those items should be reported in abstracts.

### Introduction: background and objectives

#### Item 2a

Standard CONSORT 2010 item: scientific background and explanation of rationale.

Extension for embedded recruitment trial: scientific background and explanation of rationale for the *embedded recruitment trial including a brief description of the host trial(s) as appropriate.*

Example:

Randomised controlled trials are the ‘gold standard’ for the evaluation of the effectiveness and safety of health care interventions, particularly because they protect against selection bias. However, recruiting clinicians and patients to randomised trials can be extremely difficult. Trialists use many interventions to improve recruitment, but evidence regarding the likely effect of these interventions is often unclear.

The web-based intervention modelling experiment (WIME) study (ClinicalTrials.gov number NCT01206738) has the primary aim of running a WIME to develop and evaluate theory-based interventions to improve antibiotic prescribing for upper respiratory tract infections in primary care. It also has an embedded trial evaluating which of two invitation methods, e-mail or post, is most effective at recruiting general practitioners (GPs) to the study, which is the subject of this article [11].

Explanation:

The CONSORT statement encourages authors to describe the problem that necessitated the work [18]. The need for an embedded trial of alternative recruitment approaches may have been identified prior to the host trial commencing and planned alongside the host trial (e.g. Treweek et al. [11]). Alternatively, trial methodologists interested in evaluating new recruitment approaches may collaborate with suitable host trials for embedding recruitment trials (e.g. Man et al. [13], see Additional file 1, Item 2a, example 1).

Lastly, recognition of the need for an embedded trial of different recruitment strategies may have arisen from on-going recruitment difficulties in the host trial(s) (e.g. Ford et al. [19], see Additional file 1, Item 2a, example 2). In such cases the nature, scope, and severity of the problem is an intrinsic part of the background and provides a compelling rationale for the embedded trial. Furthermore, an embedded trial is always conducted in the context of its host RCT(s). Therefore, embedded recruitment trial reports should provide sufficient information (such as population, intervention, settings and reference(s) to descriptions of host trial(s), e.g. protocol or findings) about the host trial(s) and their recruitment processes or issues to understand the context of the embedded recruitment trial.

In most recruitment trials we have used as examples for this guideline, the brief description of the host trial was given in the background. We therefore suggest that this may be the most appropriate place to locate such a description, but we acknowledge that sometimes the brief description of the host trial will sit better at a later point in the manuscript, in relation to the embedded trial setting (see item 4b).

If the embedded recruitment trial was not planned in response to specific recruitment difficulties in the host trial, authors’ rationale for the embedded trial must, nevertheless, be clearly explained (e.g. Treweek et al. [11], Man et al. [13]).

#### Item 2b

Standard CONSORT 2010 item: specific objectives or hypotheses.

Extension for embedded recruitment trial: specific objectives or hypotheses for the *embedded recruitment trial.*

Example:

To assess whether optimised patient information materials improved the proportion of patients responding positively to an invitation to take part in each trial and the proportion actually randomised [13].

Explanation:

Explanations for reporting specific objectives and hypothesis of the study clearly are discussed by Moher et al. [7].

### Methods: trial design

#### Item 3a

Standard CONSORT 2010 item: description of trial design (such as parallel, factorial) including allocation ratio

Extension for embedded recruitment trial: description of *embedded recruitment trial* design (such as parallel, factorial, *cluster*) including allocation ratio.

Example:

We used a 2 × 2 factorial design to distribute practices and participants across two trial design factors: cluster versus individual allocation and systematic versus opportunistic recruitment.

We randomly assigned 24 practices (8 practices in each of 3 geographical regions (Bristol, Devon and Coventry)) in a 3:1 ratio to cluster (practice) allocation or individual allocation, and in a 1:1 ratio to opportunistic or systematic recruitment [20].

Explanation:

These guidelines apply to all embedded recruitment trials. As many embedded trials are cluster randomised (for example, in the MRC START programme 7 out of 11 embedded recruitment trials were cluster randomised), we have added this to the list of designs given as examples in the item descriptor. For additional information required in reporting other trial designs such as cluster randomised trials, refer to appropriate CONSORT extensions.

#### Item 3b

Standard CONSORT 2010 item: important changes to methods after trial commencement (such as eligibility criteria), with reasons.

Extension for embedded recruitment trial: important changes to methods of the *embedded recruitment trial* after commencement (such as eligibility criteria), with reasons.

Example:

In a primary prevention trial with postmenopausal hormone therapy (PHT; also known as hormone replacement therapy, HRT), we wanted to study the effect on numbers recruited and the process of recruitment when using blinding (the blind group) as compared to the situation when both the caregiver and the woman will know which arm the woman is in (the non-blind group).

In the original study protocol, ultrasound examination of the uterus in the non-blind group was to be made only in the PHT arm and only after the envelope had been opened; however, physicians wanted to provide a clinical service for the women, and most women in the non-blind group were examined before the opening of the envelope [21].

Explanation:

Moher et al. [7] provides detailed explanations for reporting important changes to methods after trial commencement.

### Participants

#### Item 4a

Standard CONSORT 2010 item: eligibility criteria for participants.

Extension for embedded recruitment trial: eligibility criteria for participants for the *embedded recruitment trial, including any differences from those for the host trial(s).*

Example:

Patients were eligible for the study if they (1) had a diagnosis of colorectal, breast or lung cancer; (2) were clinically eligible for entry into a cancer treatment trial randomised against control/standard treatment, or best supportive care; (3) had access to a video recorder, CD-ROM or DVD playing facilities; and (4) could understand English [22].

Explanation:

The embedded trial eligibility criteria may or may not be identical to that for the host trial(s) (e.g. Man et al. [13], see Additional file 1, Item 4a, example 1). For example, the embedded trial may focus on a subgroup of participants for practical or other reasons (e.g. Hutchinson et al. [22]) or where recruitment to host trial(s) was a particular issue or it may include a wider group, with the view of identifying potential participants eligible for host trial(s), who will then be included in the host trial based on more restrictive inclusion criteria (e.g. Tworoger et al. [23], see Additional file 1, Item 4a, example 2). Eligibility criteria for the embedded trial therefore should be clearly defined and should explicitly state whether this did or did not differ from the host trial(s).

#### Item 4b

Standard CONSORT 2010 item: settings and locations where the data were collected.

Extension for embedded recruitment trial: settings and locations where the *embedded recruitment trial was carried out, including a brief description of the host trial(s) as appropriate.*

Example:

Those eligible for enrolment in the study were HIV-1 positive men and women attending the HIV clinic at the Chelsea and Westminster Hospital in west London between January 1997 and June 1998. Specific clinics where the physicians were involved in clinical trials and known to refer patients to trials were targeted, and all patients attending that clinic were asked if they would like to join this study [24].

Explanation:

Careful descriptions of the trial participants and the setting in which they were studied are needed so that readers may assess the external validity (generalisability) of the trial results [18]. Often settings and locations for the embedded trial are constrained by its host trial. Therefore, if a brief description of the host trial(s) setting is not provided in the introduction, this could be an appropriate place to add it (e.g. Kiernan et al. [25], Coyne et al. [26], see Additional file 1, Item 4b, examples 1–2). Such descriptions, whether here or in the introduction would normally include simple statements about the population, aim (or equivalently intervention and outcome) and reference(s) to descriptions of host trial(s).

It is important to note, however, the recruitment intervention in the embedded trial may not necessarily be conducted in the same setting as the intervention in the host trial (e.g. Ives et al. [24]). Further examples of clinical trials recruiting participants in different locations/settings to where the interventions were carried out are found in trials using media campaigns for recruitment [27, 28]. Settings and locations for the embedded trial must therefore be clearly reported.

### Interventions

#### Item 5

Standard CONSORT 2010 item: the interventions for each group with sufficient details to allow replication, including how and when they were actually administered.

Extension for embedded recruitment trial: the interventions for each group *(including control group) within the embedded recruitment trial* with sufficient details to allow replication, including how, where and when they were actually administered.

**Fig. 2:**
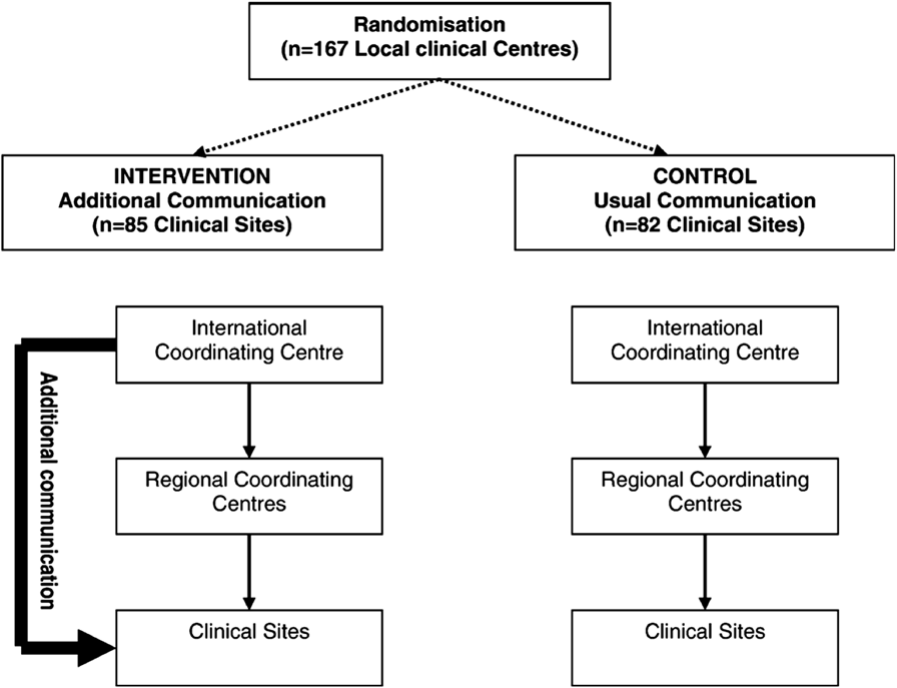
Flow chart illustrating intervention and control sites and route of additional communication strategy

Example:

Our investigation was a single-blind RCT conducted within the organisational structure established for the management of clinical sites in ADVANCE. Within this structure the usual route for communication of information was from the central trial coordinators based at the International Coordinating Centre in Sydney, Australia, to one of five Regional Coordinating Centres (Beijing, London, Melbourne, Montreal, Utrecht) who then passed on information to the clinical sites (Fig. 2). This structure included little direct communication between the central trial coordinators in Sydney and the clinical sites, and it was the effect of instituting additional communication between central coordinators and the clinical centres that was evaluated in the trial reported here.

Control—usual communications

Usual communications to the clinical sites were provided via the Regional Coordinating Centres with the International Coordinating Centre providing only occasional direct communications in the form of generic newsletters, e-mails and faxes. The Regional Coordinating Centres provided routine monitoring activities, which included frequent telephone, e-mail and personal contacts to support recruitment activities.

Intervention—additional communications.

The additional communication strategy was applied on top of the usual communication strategy. The additional communication strategy from the central trial coordinators to the clinical sites involved frequent e-mail contact, regular personalised mail-outs of league tables and graphs describing recruitment performance relative to other centres, individualised certificates acknowledging achievement of recruitment milestones, and items related to the study (e.g. an ‘ADVANCE computer mouse-mat’). E-mails to the clinical sites from the central trial coordinators generally contained highly-tailored site-specific information about recruitment performance relative to goals, along with messages of support and encouragement. On average, sites assigned to the intervention received about one of these additional pieces of communication intended to enhance recruitment from the International Coordinating Centre each month [29].

**Fig. 3:**
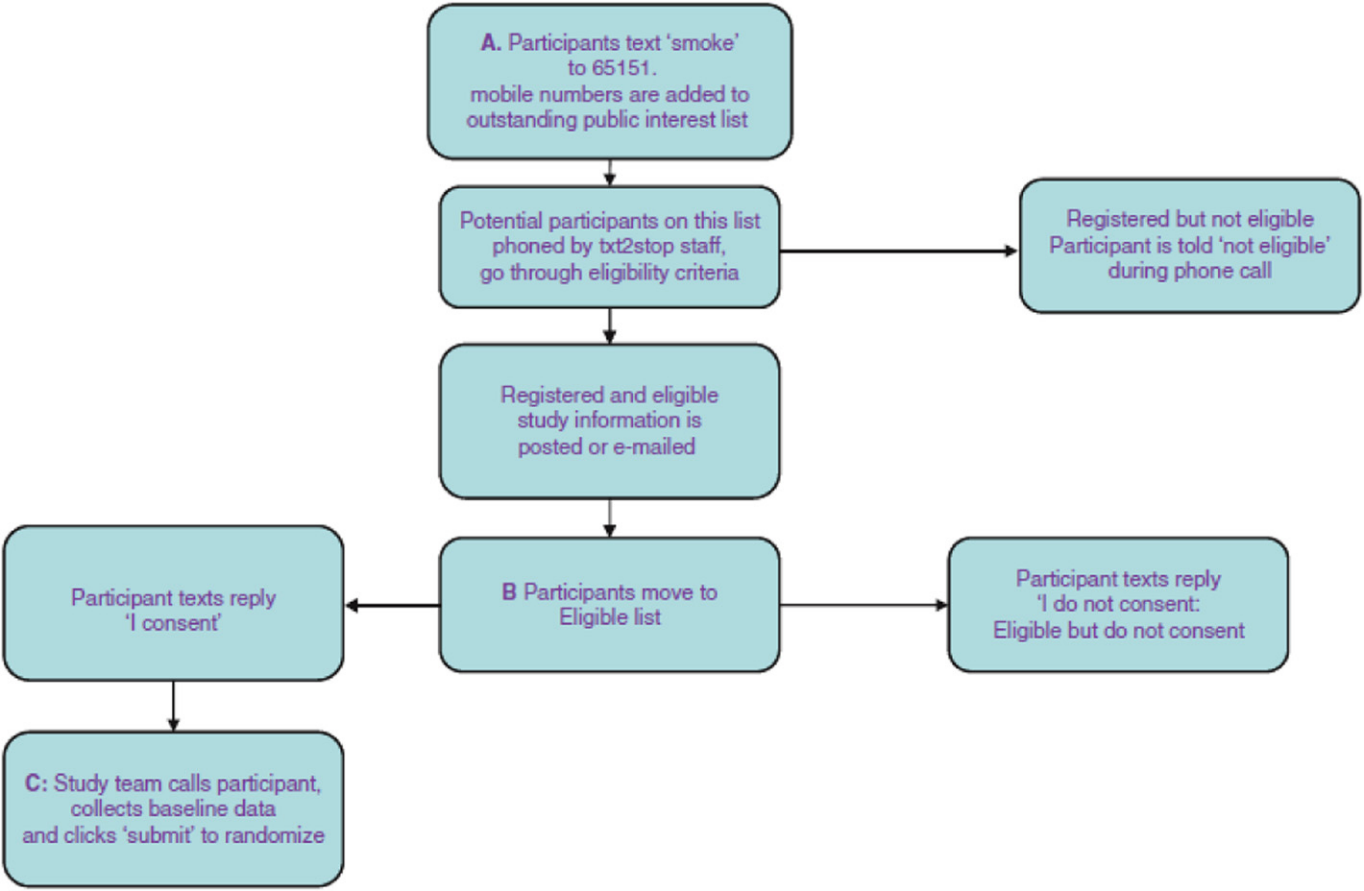
A flow chart illustrating the focal point(s) of the host trial recruitment pathway targeted by embedded recruitment trial interventions

Explanation:

It is important to note that interventions in the embedded trial are always directed towards a host trial(s). Some embedded trial interventions may test different trial designs (illustrated in Avenell et al. [30], see Additional file 1, Item 5, example 1). Others may focus on trial procedures, for example, Monaghan et al. [29] evaluated the effects of different communication procedures between central coordinators and the clinical sites on participant accrual. Therefore, authors should clearly report the aspect of the host trial(s) (i.e. procedures or design itself) targeted by the recruitment intervention in the embedded trial. It is strongly recommended that the focal point in the host trial targeted by recruitment intervention is illustrated in a flow chart. This could be part of the CONSORT flow chart for the host trial, but often it will be easier to illustrate in a separate flow chart (Monaghan et al. [29], Fig. 2 and Free et al. [31], see Additional file 1, Item 5, example 2; Fig. 3).

The description of the recruitment intervention may include details such as form and content, format and, if applicable, any extra features such as incentives, timing of delivery, methods of reminders/follow-up and how undelivered invitations were treated. A template for reporting intervention descriptions is provided in Hoffmann et al. [32].

### Outcomes

#### Item 6a

Standard CONSORT 2010 item: completely defined pre-specified primary and secondary outcome measures, including how and when they were assessed.

Extension for embedded recruitment trial: completely defined pre-specified primary and secondary outcome measures for the *embedded recruitment trial,* including how and when they were assessed.

Example:

The primary study end point was the percentage of patients in the study who declined participation in the clinical trial that was presented to them.

Data were collected from patient case notes and directly from questionnaires. Patients were seen on two occasions for the purposes of this trial. These visits were part of patients’ general medical care, as they were attending the hospital anyway on these days. These were known as ‘visit 1’ (explanation of treatment trial) and ‘visit 2’ (return visit, usually 1 week later, to discuss their decision). Demographic data on all patients who were approached for the AVPI study were collected and entered into a log sheet [22].

Explanation:

Primary and secondary outcome measures for embedded recruitment trials must be considered separately from the host trial(s). Types of outcome may include acceptance and decline rates (e.g. Hutchinson et al. [22]), recruitment rates, retention rates, time to complete recruitment, consenting rates, quality of informed consent and time and cost incurred. It is important to note that acceptance and decline rates are not necessarily the same outcome with direction reversed. For example, to be considered as a valid acceptance or decline of the invitation (i.e. recruitment intervention), patients may be required to return an acceptance or decline form actively. In this case, non-responders do not fulfil the pre-defined criteria for a valid acceptance nor a decline of the invitation (e.g. Man et al. [13], see Additional file 1, Item 6a). For each outcome, the definitions for denominators must be clearly reported and for rates, numerators must also be clearly reported.

It is important to consider that the primary and secondary end points of an embedded recruitment trial do not generally coincide with those of the host trial (e.g. Hutchinson et al. [22]). For readers to be able to assess outcomes reliably, information on the data collection process (what was collected, when and how) for the embedded recruitment trial must be clearly reported.

#### Item 6b

Standard CONSORT 2010 item: any changes to trial outcomes after the trial commenced, with reasons.

Extension for embedded recruitment trial: any changes to the *embedded recruitment* trial outcomes after the *embedded recruitment trial* commenced, with reasons.

Example:

In the Healthlines Depression recruitment trial, the primary outcome was the proportion of patients randomised. Secondary outcomes were the proportion of patients who accepted the offer of invitation to participate, and the proportion of eligible patients who actively opted out of the trial (i.e. returned a ‘decline’ form).

In the Healthlines CVD recruitment trial, the primary outcome was the proportion of patients who responded positively to the invitation to participate. This, rather than actual randomisation, was selected as the primary outcome because of a cap on recruitment numbers, whereby only the first 25 eligible participants were randomised in each practice. This upper limit was implemented because of practice staff availability to carry out these assessments, and an initial agreement with researchers that 25 patients would be sufficient to reach target recruitment across participating GP practices. The secondary outcome was the proportion of eligible patients who actively opted out [13].

Explanation:

A detailed explanation for reporting important changes to methods after trial commencement was provided by Moher et al. [7].

#### Item 7a

Standard CONSORT 2010 item: how sample size was determined.

Extension for embedded recruitment trial: how sample size for the *embedded recruitment trial* was determined.

Example:

This was a pragmatic trial, so we included all participants on the ‘outstanding public interest list’ for the Txt2stop trial. In June 2008 there were 937 potential participants on the ‘outstanding public interest’ list. A sample size of 937 gives a 90 % chance of detecting an absolute difference of 4.5 % in registrations (6.5 % in the intervention group compared to 2 % in the control group) at a two-sided alpha = 0.05 [31].

Explanation:

It should be noted that in many embedded trials of recruitment interventions, the numbers of eligible patients potentially available for the embedded trial may be far larger than the sample size calculated for the host trial. For example, if a trial recruiting 500 patients estimates a 10 % participation rate, there may be 5000 eligible patients for the embedded trial.

However, because of the restrictions posed by their host trial(s), it is not necessarily appropriate for embedded trials to estimate and set a target sample size capable of detecting the minimally important difference in its primary outcome. For example, if the number of participants required for the host trial is known, then the maximum number of patients that the embedded trial can approach is as many eligible participants as it takes to recruit that number (although the number approached may be smaller). Here, performing a sample size calculation for number of participants to be approached in the embedded trial is not meaningful. In such cases, authors should clearly report how the sample size has been determined (e.g. Treweek et al. [11], see Additional file 1, Item 7a, example 1). However, it is usually possible to report the expected sample size and effect sizes that are detectable at 80 % or 90 % power for the embedded trial, based on host trial(s) requirements, if certain assumptions are made (e.g. Free et al. [31]). Where formal sample size calculations have been performed, these should be reported (e.g. Hutchinson et al. [22], see Additional file 1, Item 7a, example 2).

#### Item 7b

Standard CONSORT 2010 item: when applicable, explanation of any interim analyses and stopping guidelines

Extension for embedded recruitment trial: when applicable, explanation of any interim analyses and stopping guidelines for the *embedded recruitment trial*

We have not included examples or explanations for this item as there are no additional recommendations made specifically relevant for embedded recruitment trials. Rationale for reporting this item is provided by Moher et al. [7].

### Randomisation: sequence generation

#### Item 8a

Standard CONSORT 2010 item: method used to generate the random allocation sequence.

Extension for embedded recruitment trial: method used to generate the random allocation sequence for the *embedded recruitment trial.*

Example:

A de-identified list of eligible participants (that is a list of unique study numbers corresponding to the eligible participants but containing no personal identifiers) was prepared by the research nurse. To ensure concealment of allocation, one of the investigators (JJY), who was not directly involved in the recruitment of the patients from family physicians’ offices and who was unaware of the patients’ identities, produced a computer-generated randomisation sequence (block size 4) stratified by family practice. This investigator (JJY) then allocated each unique study number to either telephone or mail follow-up (in a 1:1 ratio) according to the randomisation sequence and returned the participant allocation list to the research nurse [17].

Explanation:

Detailed explanations on why this item should be reported clearly were given in Altman et al. [18] and Moher et al. [7].

#### Item 8b

Standard CONSORT 2010 item: type of randomisation; details of any restriction (such as blocking and block size)

Extension for embedded recruitment trial: type of randomisation; details of any restriction (such as blocking and block size) in the *embedded recruitment trial.*

Example:

Patients were first stratified on the basis of age and type of randomised clinical trial for which consent was sought: trials comparing treatment with no treatment; trials comparing dissimilar treatments; and trials comparing similar treatments [33].

Explanation:

As for any RCT, in embedded trials a sensible evaluation and choice of stratification/minimisation factors is called for (e.g. Avenell et al. [30], see Additional file 1, Item 8b). Where the host trial is using cluster randomisation, and the embedded trials also use cluster randomisation, it is important that the allocation of the recruitment intervention is balanced over the host clusters. There may be a risk of unbalanced allocation where the number of clusters is small, and any mechanisms used to ensure balance should be described. If the recruitment trial is embedded in more than one host trial, the host trial itself could be an important factor influencing recruitment outcomes. For an example, the baseline recruitment rates across different patient populations included in host trials may differ. Similarly, the responses for embedded recruitment intervention may differ because of the differential acceptability of the recruitment interventions and other trial-specific aspects of the design in different host trials. Where there are multiple host trials, therefore, the host trial should be considered as a possible stratification or minimisation factor for embedded trials, e.g. Simes et al. [33].

### Allocation concealment mechanism

#### Item 9

Standard CONSORT 2010 item: mechanism used to implement the random allocation sequence (such as sequentially numbered containers), describing any steps taken to conceal the sequence until interventions were assigned) [49].

Extension for embedded recruitment trial: mechanism used in the *embedded recruitment trial* to implement the random allocation sequence (such as sequentially numbered containers), describing any steps taken to conceal the sequence until interventions were assigned.

### Example:

A randomisation list was generated using a random numbers table and held centrally at the Data Coordinating Centre. The research nurse obtained each participant’s allocation assignment by phone from a member of the study staff [34].

### Explanation:

Altman et al. [18] and Moher et al. [7] provide detailed explanations for including this item.

### Implementation

#### Item 10

Standard CONSORT 2010 item: who generated the random allocation sequence, who enrolled participants, and who assigned participants to interventions?

Extension for embedded recruitment trial: who generated the random allocation sequence(s), who enrolled participants, and who assigned participants to *embedded recruitment* interventions?

Example:

The study statistician (GM) generated a list of random numbers and participant IDs broken down into mailing blocks, which SPCRN used to randomly allocate GPs to receive either an e-mail or a postal invitation on a 1:1 basis without stratification [11].

Explanation:

Altman et al. [18] and Moher et al. [7] provide detailed explanations for reporting this item.

### Blinding

#### Item 11a

Standard CONSORT 2010 item: if done, who was blinded after assignment to interventions (for example, participants, care providers, those assessing outcomes) and how?

Extension for embedded recruitment trial: if done, who was blinded after assignment to *embedded recruitment interventions* (for example, participants, care providers, those assessing outcomes) and how?

Example:

Although participants were aware of their group status (i.e. whether or not they received a telephone call or a questionnaire), they were unaware of the other groups’ status and that recruitment into the study was being monitored on the basis of this, as part of a RCT. It was impractical for the research nurse administering the interventions (posting out questionnaires and contacting people by telephone) and assessing the outcome (recruitment into physical activity study) to be blinded to their group status [35].

Explanation:

In controlled trials, the term blinding usually refers to keeping study participants, health care providers, and sometimes those collecting and analysing clinical data unaware of the assigned intervention, so that they will not be influenced by that knowledge [18]. Blinding means more than just keeping the name of the treatment hidden. In drug trials, ‘patients may well see the treatment being given to patients in the other treatment group(s), and the appearance of the drug used in the study could give a clue to its identity. Differences in taste, smell, or mode of delivery may also influence efficacy, so these aspects should be identical for each treatment group. Even colour of medication has been shown to influence efficacy. In studies comparing two active compounds, blinding is possible using the “double dummy” method. For example, if we want to compare two medicines, one presented as green tablets and one as pink capsules, we could also supply green placebo tablets and pink placebo capsules so that both groups of patients would take one green tablet and one pink capsule’ [36]. In recruitment trials, however, both participants and providers are aware of the intervention they receive/deliver.

For example, in an embedded recruitment trial testing two types of invitation letters, both participants and intervention providers have the full knowledge of what they had (i.e. wording, format, lettering, and colour of the invitation letter). Therefore, it is not possible to keep them blind to the intervention (i.e. to keep what treatment group they are being assigned to hidden from them). In these situations, researchers may consider blinding the participants/providers to the existence of other treatment arms or to the embedded trial altogether. This was the case in the Harris et al. [35], for example.

Thus, while information that is often withheld in placebo-controlled trials, about which treatment the participants are getting cannot be withheld, information that is often given to participants about the trial and arms other than those they are allocated to is withheld.

Furthermore, consent to participate is extremely unlikely in these trials since it does not usually make any sense to ask individuals if they would like to take part in a trial of recruitment strategies—this would not make the results of the trial relevant to real practice and would probably result in reduced recruitment rates, the very opposite of the recruitment RCT aim.

This withholding of information is, in some sense, an alternative form of blinding and in embedded recruitment trials: therefore, to assess whether participant/provider blinding was achieved in any meaningful way, the information given to participants/providers about the embedded trial must be clearly reported. For example, Harris et al. [35], Lienard et al. [37] and Monaghan et al. [29] (see Additional file 1, Item 11a, examples 1–2) reported clear details about the information that was given to or withheld from those approached.

#### Item 11b

Standard CONSORT 2010 item: if relevant, description of the similarity of interventions.

Extension for embedded recruitment trial: if relevant, description of the similarity of interventions in the *embedded recruitment trial.*

Example:

Both units received identical literature about the study, received the same slide presentation on the study protocol, and had the same opportunity to ask questions. This scripting was designed to ensure consistency between units and minimise potential sources of bias [38].

Explanation:

In any RCT, for successful blinding and therefore elimination of bias, it is important that all elements of the trial except the intervention that is being evaluated are kept the same across all arms. Further, this will ensure that the outcome(s) of interest are not unduly influenced by other trial aspects (for example environment of the room where interventions administered). While blinding in the usual sense may not be possible in embedded recruitment trials (see item 11a) it is still important that apart from the active interventions, all other aspects of the intervention are similar in order to avoid bias. For example, in Fowell et al. [38] the active interventions were cluster randomisation versus randomised consent but other aspects were identical in both arms.

### Statistical methods

#### Item 12a

Standard CONSORT 2010 item: statistical methods used to compare groups for primary and secondary outcomes.

Extension for embedded recruitment trial: statistical methods used to compare groups for primary and secondary outcomes of the *embedded recruitment trial.*

Example:

Outcomes were first described separately by arm, and then compared using logistic regression to estimate the between-group odds ratio (OR) and corresponding 95 % confidence interval (CI) on the basis of the intention-to-treat principle [13].

Explanation:

Rationale for reporting statistical methods used to compare groups is given by Altman et al. [18] and Moher et al. [7].

#### Item 12b

Standard CONSORT 2010 item: methods for additional analyses, such as subgroup analyses and adjusted analyses.

Extension for embedded recruitment trial: methods for additional analyses, such as subgroup analyses and adjusted analyses for the *embedded recruitment trial.*

Example:

The economic evaluation was conducted from the perspective of the UK NHS. Data gathered were the duration of the visit and the grade of recruitment staff. Staff time was valued using annual salaries, including employer oncosts (UK statutory contributions to pensions and national insurance), obtained from one centre, adjusted for number of weeks worked per year, number of hours worked per week, and the proportion of time spent with patients. Comparisons between nurses and urologists were performed using *t* tests and 95 % CIs for differences between means. Planned sensitivity analyses were undertaken to explore the impact of the number of appointments and staff present [39].

Explanation:

Embedded trials assessing approaches to recruitment are of particular interest to researchers looking to apply these methods to their own trials. For researchers, not being able to recruit to target on time can delay the study while increasing cost [1, 40]. In some trials, recruitment issues may contribute to eventual stopping of the trial mid-way [41]. Reports of recruitment interventions must therefore strive to provide as much information as possible on any additional analyses done (e.g. the analyses of gender differences in the paper by Man et al. [13], see Additional file 1, Item 12b) for intended users, so that they can make an informed decision. Recruitment cost is an important aspect of any trial budget. Where possible, reports on recruitment interventions should therefore include an economic evaluation as an integral part of their analysis (e.g. Donovan et al. [39]). This may not necessarily be a full health economic analysis. Further, we suggest that if effects on retention are not evaluated as primary or secondary outcome(s), trials of recruitment interventions should consider including retention as a long-term outcome. These could be classed as additional analyses.

### Results: participant flow (a diagram is strongly recommended)

#### Item 13a

Standard CONSORT 2010 item: for each group, the numbers of participants who were randomly assigned, received intended treatment, and were analysed for the primary outcome.

Extension for embedded recruitment trial: for each group in the *embedded recruitment trial,* the numbers of participants who were randomly assigned, received intended treatment, and were analysed for the primary outcome.

**Fig. 4:**
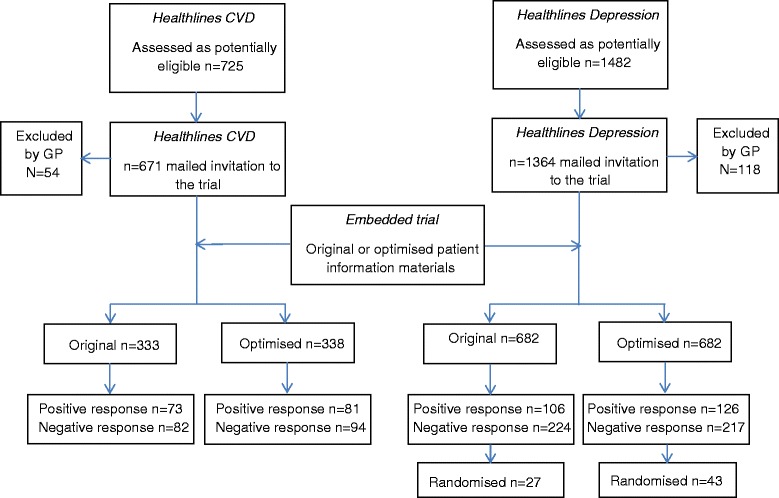
Recruitment flowchart

Example:

Figure 4 presents the participant flowchart for an embedded trial where recruitment intervention was embedded in two host trials [13].

Explanation:

Participant flow in an embedded recruitment trial will usually not coincide with its host trial(s). In some instances, the embedded trial could be included as an integral part of the host participant flow chart. In others, the embedded trial may assess the numbers randomised to the host trial(s) as its primary outcome and therefore could be viewed as an appendix to the host trial(s) flow chart. The flow chart should provide information on how many were allocated to each group in embedded trial, whether all participants did or did not receive the allocated treatment and, if known, why some did not, or exclusions from analysis. In embedded recruitment trials there are technically no losses to follow-up since it will be known for each participant approached whether they were recruited or not. Nevertheless, investigators may wish to distinguish between those who refused and those who did not respond.

**Fig. 5:**
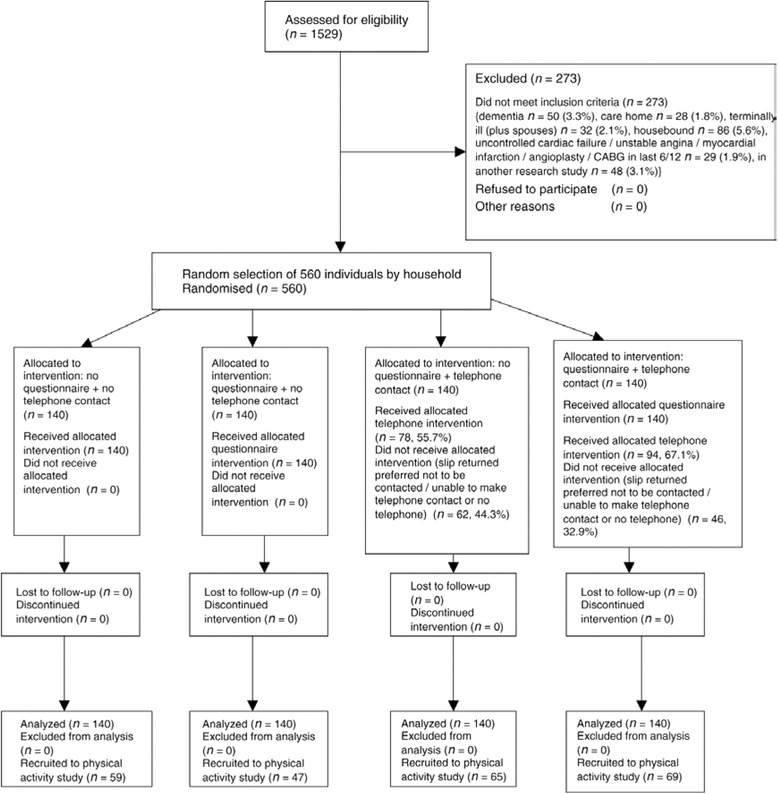
Recruitment flowchart

**Fig. 6:**
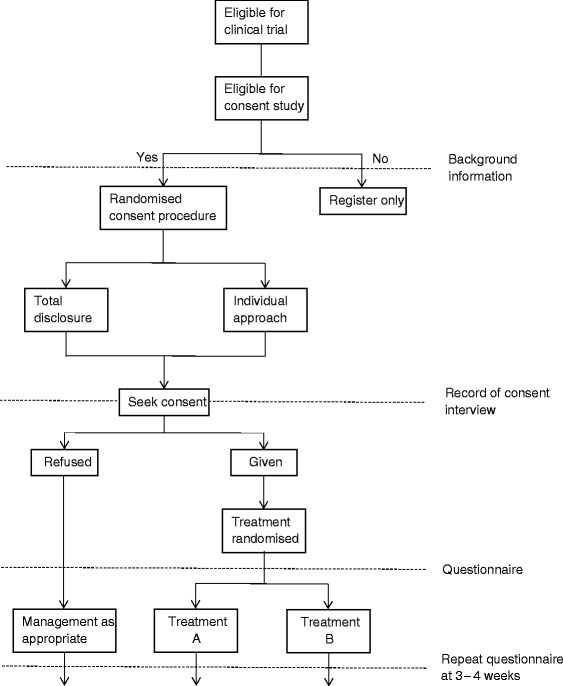
Recruitment flowchart

The exact layout and content of the flow chart is trial dependent, and to a certain extent will depend on the respective host trial(s) and characteristics of recruitment interventions. For example, the information presented in the flow chart may include overall numbers approached, accepted, did not respond, undelivered, declined, recruited and randomly assigned, received intended recruitment intervention and retained with full follow-up as appropriate, and were included in primary outcome analysis of host trial(s). Examples of different layouts that could be used for presenting embedded trials are provided (Figs. 4, 5 and 6).

#### Item 13b

Standard CONSORT 2010 item: for each group, losses and exclusions after randomisation, together with reasons).

Extension for embedded recruitment trial: for each group, losses and exclusions after randomisation to the *embedded recruitment trial,* together with reasons.

**Fig. 7:**
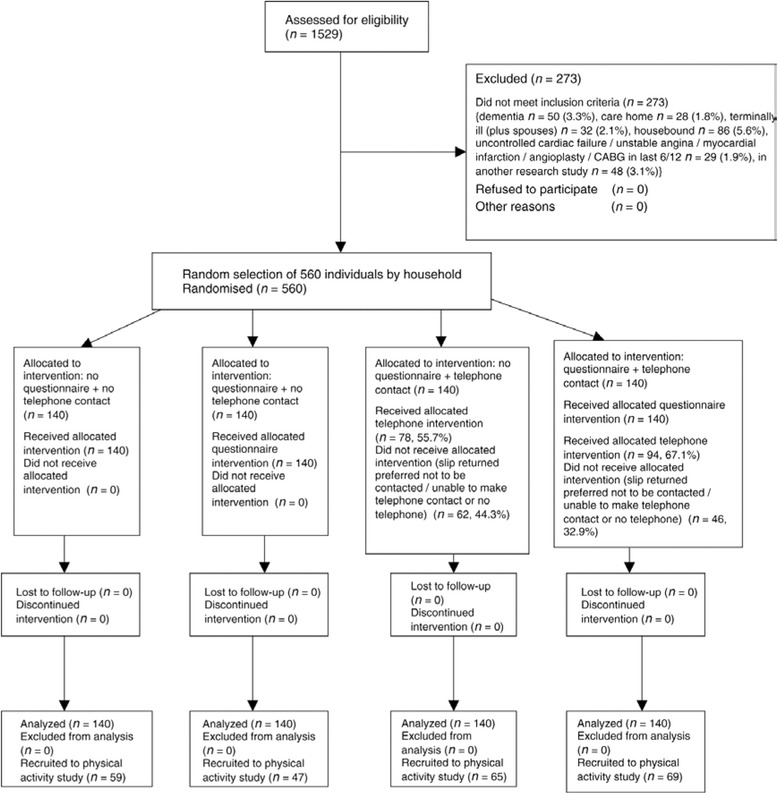
CONSORT diagram showing the flow of participants through each stage of the randomised trial (enrolment, intervention allocation, follow-up and data analysis)

Example:

Of the 1529 patients aged ≥65 years registered with the practice, 273 (17.9 %) were excluded. See Fig. 7 for details [35].

Explanation:

The intervention in an embedded recruitment trial is usually short in nature and losses during the intervention period are therefore unlikely. Exclusions may happen between randomisation and the intervention (e.g. Harris et al. [35]).

#### Item 14a

Standard CONSORT 2010 item: dates defining the periods of recruitment and follow-up.

Extension for embedded recruitment trial: dates defining the periods of recruitment and follow-up for *both embedded recruitment trial and host trial(s).*

Example:

The intervention study is investigating the effect of a 1-year moderate intensity exercise intervention on serum levels of endogenous sex hormones in postmenopausal women.

Recruitment began in January 1998 and finished in July 2000. Previous research suggests that different mailing strategies can affect the response rate. Therefore, at the beginning of the recruitment period, we conducted a pilot study of four mailing strategies and used the results to determine the mailing method for use during the remainder of recruitment. The recruitment packets for the pilot study were mailed 3 weeks apart in February and March 1998 [23].

Explanation:

It is important to note that the embedded recruitment trial may not necessarily start nor will it necessarily continue for the duration of the host trial(s). Therefore, authors should clearly report whether the embedded recruitment trial lasted as long as the host trial, or was shorter.

#### Item 14b

Standard CONSORT 2010 item: why the trial ended or was stopped.

Extension for embedded recruitment trial: why the *embedded recruitment trial* ended or was stopped.

Example:

The present study was carried out within a randomised clinical trial for patients with node-positive breast cancer (AERO-B2000). The present study initially aimed at studying the impact of all monitoring visits, i.e. initiation, on-going and closeout visits on (1) patients’ recruitment, (2) quantity of data spontaneously reported, (3) quality of data, and (4) patients’ follow-up time.

The study started in March 2000 and was terminated in March 2002, when the AERO group decided to redirect on-site monitoring visits (which is the recruitment intervention evaluated in this trial) to centres in which a problem had been identified, either because they had not sent any data to the coordination centre, or because they lacked time and adequate administrative support to complete the case report forms. This shift in monitoring activities implied termination of the present study, with most initiation visits completed as planned but only a few on-site visits and no closeout visits [37].

Explanation:

Moher et al. [7] discussed the reasons for reporting this item in detail.

### Baseline data

#### Item 15

Standard CONSORT 2010 item: a table showing baseline demographic and clinical characteristics for each group.

Extension for embedded recruitment trial: *if possible* a table showing baseline characteristics of *each arm of the embedded recruitment trial.*

Example:

Table 2 shows an example where researchers were able to collect baseline characteristics from participants approached [42].

**Table 2:** Characteristics of all participants (*n* = 703) [42]

	Intervention	Control
	(*n* = 356)	(*n* = 347)
Mean age	41.11	41.53
	(SD = 10.9)	(SD = 10.7)
Gender		
Male	116 (32.6 %)	104 (30.0 %)
Female	240 (67.4 %)	243 (70.0 %)
Number of episodes of sick leave		
One episode in the last 2 years	168 (47.2 %)	179 (51.6 %)
Two or more episodes in the last 2 years	188 (52.8 %)	168 (48.4 %)
Diagnosis		
Mental health problems	163 (45.8 %)	160 (46.1 %)
Muscle-skeletal problems	154 (43.3 %)	147 (42.4 %)
Other diagnosis	39 (11.0 %)	40 (10.5 %)

Explanation:

In recruitment trials it is the participants approached who should be included in the baseline table. It is not always possible to know the number of eligible participants approached or their characteristics (illustrated in Additional file 1, Item 15, example 2 [43]: in this trial the focal point for the recruitment intervention was telephone screening of participants for eligibility).

### Numbers analysed

#### Item 16

Standard CONSORT 2010 item: for each group, number of participants (denominator) included in each analysis and whether the analysis was by original assigned groups.

Extension for embedded recruitment trial: for each group in the *embedded recruitment trial,* number of participants (denominator) included in each analysis and whether the analysis was by original assigned groups.

Example:

A total of 2397 families were randomised, 1203 to receive the questionnaire with the invitation to take part and 1194 to receive the invitation without the questionnaire. Four invitations were returned as not known at that address and were excluded from the analysis [44].

Explanation:

The number of participants in each intervention arm is an essential element of RCT results. Failure to include all participants may bias trial results [18]. Denominator data for embedded trials tends to be poorly reported. It is important to note that in embedded recruitment trials, the denominator is usually the number of participants who were initially approached. This is a larger number than that used in power calculations for clinical outcomes within individual host trial(s). In some recruitment interventions (such as newspaper advertisements), however, this number is impossible to know (e.g. Miller et al. [43], see Additional file 1, Item 16: in this trial the numbers who responded to media advertisements were included as the denominator).

### Outcomes and estimation

#### Item 17a

Standard CONSORT 2010 item: for each primary and secondary outcome, results for each group, and the estimated effect size and its precision (such as 95 % CI) [50].

Extension for embedded recruitment trial: for each primary and secondary outcome, results for each group in the *embedded recruitment trial,* and the estimated effect size and its precision (such as 95 % CI).

Example:

Telephone contact by the research nurse increased the recruitment rate: contact 47.9 % (134/280), no contact 37.9 % (106/280) and difference 10.0 % (95 % CI 0.2–19.8) (Table 3) [35].

**Table 3:** Effect of interventions on recruitment into host trial [35]

Interventions	Recruitment in intervention group	Recruitment in control group	Difference in recruitment intervention minus control (95 % CI) adjusted for the clustering effect of household (395 households)	OR (95 % CI) for recruitment into study adjusted for clustering effect of household (395 households)
Telephone (groups 3 + 4 versus control (groups 1 + 2)	134/280 (47.9 %)	106/280 (37.9 %)	10.0 % (0.2–19.8 %)	1.5 (1.0–2.3)
				*P* = 0.046
Questionnaire (groups 2 + 4) versus control (groups 1 + 3)	116/280 (41.4 %)	124/280 (44.3 %)	−2.9 % (−12.7–7.0 %)	0.9 (0.6–1.3)
				*P* = 0.570

Explanation:

Rationale for reporting this item is given in Moher et al. [7].

#### Item 17b

Standard CONSORT 2010 item: for binary outcomes, presentation of both absolute and relative effect sizes is recommended

Extension for embedded recruitment trial: for binary outcomes in the *embedded recruitment trial*, presentation of both absolute and relative effect sizes is recommended.

Example:

Of the 180 people given information about the open trial design, 134 (74.4 %) consented, compared with 233 (65.1 %) of 358 given information about the blinded, placebo-controlled design (difference 9.4 %, 95 % CI 1.3–17.4 %; OR 1.56, 95 % CI 1.05–2.33). The OR was not materially influenced by adjusting for age, sex, type of fracture and time since fracture (OR 1.58, 95 % CI 1.06–2.36) [30].

Explanation:

An explanation for reporting this item is provided in Moher et al. [7].

### Ancillary analyses

#### Item 18

Standard CONSORT 2010 item: results of any other analyses performed, including subgroup analyses and adjusted analyses, distinguishing pre-specified from exploratory

Extension for embedded recruitment trial: results of any other analyses performed for the *embedded recruitment trial*, including subgroup analyses and adjusted analyses, distinguishing pre-specified from exploratory

Example:

Using e-mail did not improve recruitment (risk difference 50.7 %, 95 % CI 2.7–4.1 %).

Sending out postal invitations and reminders took approximately two working days longer (40 vs. 26 h) than sending out e-mails. The total amount of time spent sending out the invitations and reminders was 66 h, the cost of which was estimated at approximately £1122 assuming mid-point grade 6 on the UK university pay scale. Apart from staff time, the cost of sending out e-mails was considered to be free to the WIME. The cost of materials and postage for sending postal invitations and reminders was estimated to be £1391. Our overall response rate of 15 % for postal invitations meant that the cost of sending out each round of reminders was less than the cost of the initial invitation but not dramatically so. We recruited 66 GPs from the initial 880 who received a postal invitation, meaning we sent out 814 first reminders. We received 35 responses to the first reminder and sent out 779 second reminders. The cost per reminder round of about £669 is 91 % of the £733 initial cost, which corresponds reasonably well with the fact that we re-sent materials to 93 % of GPs in the first reminder round and 89 % in the second round. The total cost of the e-mail invitations was £442 compared with £2071 for the postal invitations, giving a cost per recruit of £3.20 for e-mail and £15.69 for post. The total cost of sending the vouchers was estimated to be £371 (14 h of staff time costing £238 plus £133 printing and post). The cost to the project of getting vouchers was their face value plus some of ST’s time, estimated at around 4 h [11].

Explanation:

An example economic evaluation that authors may consider performing is presented.

### Harms

#### Item 19

Standard CONSORT 2010 item: all important harms or unintended effects in each group (for specific guidance see CONSORT for harms [45]).

Extension for embedded recruitment trial: all important harms or unintended effects in each group *for both the embedded recruitment trial and host trial(s)* (for specific guidance see CONSORT for harms [45]).

Example:

We found that monetary incentives had a positive effect on adolescents’ response to a mailed survey on their willingness to be contacted about a future smoking prevention and cessation intervention. Although response rates differed by group, use of monetary incentives did not appear to strongly bias study recruitment either toward or away from smokers, compared to no incentive, nor did they appear to bias response in terms of age or gender. However, monetary incentives did nothing to alleviate existing age and gender differences in response.

Although monetary incentives of the types employed in this study were effective in improving response rates to mail-based surveys of adolescents, with no adverse impact on willingness to further participate in intervention activities, monetary incentives are not a panacea. The observed age and gender differences in response suggest that, while manipulation of mailing strategies and use of monetary incentives may yield relatively high response rates, these techniques may not overcome differential non-response by subgroups. Moreover, beyond the initial survey response, the proportion of subjects available for subsequent randomisation into intervention study was still low, ranging from 29 % to 44 %, depending on incentive group. While we do not observe any negative differentials in willingness to be subsequently contacted, there was a fairly uniform drop-off across groups, with roughly one third of subjects who completed the survey indicating that they would not participate further. We will not know the true effects of incentives on actual study participation until follow-up, 2 years from baseline, since willingness to be contacted is only a proxy for actual participation. It remains to be seen whether those who agreed to contact and were randomised to the smoking prevention and cessation intervention will actually become engaged with the intervention activities, and whether this engagement differs by the incentive group at baseline. Finally, since the use of incentives may have established an expectation of similar incentives accompanying follow-up surveys, or otherwise altered a subject’s commitment to participate in the intervention, it remains to be seen whether there will be differential loss to follow-up between subjects who received incentives with rates at baseline and those who did not [46].

Explanation:

Readers need information about the harms as well as the benefits of interventions to make rational and balanced decisions. The existence and nature of adverse effects can have a major impact on whether a particular intervention will be deemed acceptable and useful [7]. In embedded recruitment trials, any harmful effects of embedded interventions can directly affect the host trial(s). Therefore, unintended effects in both embedded and host trial(s) must be clearly reported.

### Discussion: limitations

#### Item 20

Standard CONSORT 2010 item: trial limitations, addressing sources of potential bias, imprecision, and, if relevant, multiplicity of analyses.

Extension for embedded recruitment trial: *embedded recruitment trial* limitations, addressing sources of potential bias, imprecision, and, if relevant, multiplicity of analyses.

Example:

The major limitation of the present study is that it was terminated after 2 years, while patient recruitment was still on-going, with most centres in the visited group having been visited only once (initiation visit).

The study could not evaluate the impact of repeated on-site visits on the outcomes of interest, in particular on clinical outcomes [37].

Explanation:

The importance of reporting both pros and cons of the study was discussed by Altman et al. [18] and Moher et al. [7].

### Generalisability

#### Item 21

Standard CONSORT 2010 item: generalisability (external validity, applicability) of the trial findings.

Extension for embedded recruitment trial: generalisability (external validity, applicability) of the *embedded recruitment trial* findings.

Example:

Another drawback of this study was that more than 90 % of the participants were white women. Further studies should substantiate the study results in men and minority populations [26].

Explanation:

Rationale for discussing the generalisability of the trial finding is provided by Altman et al. [18] and Moher et al. [7].

### Interpretation

#### Item 22

Standard CONSORT 2010 item: interpretation consistent with results, balancing benefits and harms, and considering other relevant evidence.

Extension for embedded recruitment trial: interpretation consistent with results of the *embedded recruitment trial*, balancing benefits and harms, and considering other relevant evidence.

Example:

The overall physical activity study recruitment rate was 43 % (240/560) and the questionnaire survey response rate was 69 % (192/280). The latter compares well to other physical activity surveys among older primary care patients (46 %, 57 %). Our physical activity study involved participants wearing activity monitors and keeping activity logs for 1 week, less commitment than an intervention study, but more than a questionnaire survey; this is reflected in our recruitment rate being higher than physical activity intervention studies in this age group.

We found that telephone contact with a research nurse after receiving study information increased recruitment. Researchers planning studies where recruitment may be low are most likely to consider the extra costs in terms of time and effort incurred by telephoning, a valuable investment for improved recruitment. It is important to recognise that such telephone contact could be considered intrusive and an opportunity for people to opt out should be given [35].

Explanation:

An important aspect of any trial reporting is providing as much information as possible so that future studies can benefit from its findings. Therefore, where possible, interpretation of results should reflect a balanced view of benefits considering the cost and time incurred, possible mechanisms for observed differences, participant acceptability, feasibility and implications for future studies.

### Other information: registration

#### Item 23

Standard CONSORT 2010 item: registration number and name of trial registry.

Extension for embedded recruitment trial: registration number and name of trial registry *(for all host trials and embedded recruitment trial if available).*

Explanation:

Moher et al. [7] provides a detailed discussion on why this item should be reported. It is important to note that embedded methodology trials such as embedded recruitment trials may be registered as a sub-study of its host trial. If so, that information should be clearly reported.

### Protocol

#### Item 24

Standard CONSORT 2010 item: where the full trial protocol can be accessed, if available.

Extension for embedded recruitment trial: where the *embedded recruitment trial* protocol can be accessed, if available.

Explanation:

Explanation for reporting this item is given by Moher et al. [7].

### Funding

#### *Item 25*

Standard CONSORT 2010 item: sources of funding and other support (such as supply of drugs), role of funders.

Extension for embedded recruitment trial: for *embedded recruitment trial*, sources of funding and other support, role of funders *and collaborators.*

Explanation:

Supply details for new technologies or incentives used for recruitment (e.g. apps, information sheets, monetary incentives type, website addresses used as interventions) should be stated here.

## Discussion and conclusions

We have developed a revised checklist for reporting embedded recruitment trials in line with the CONSORT statement 2010. We recommend that authors should follow the principles laid out in the CONSORT statement 2010 and report all items listed as essential. Authors must pay special attention to specific recommendations we have made, where reporting requirements for these trials differ from CONSORT. The examples we have provided are intended to assist authors to report their own trials transparently. We recommend that trialists consider the embedded recruitment trial as a separate trial from its host trial(s), with all aspects of good trial design, conduct and reporting adhered to.

We have followed a robust process endorsed in EQUATOR (Enhancing the Quality and Transparency of health Research) guidelines in developing our reporting criteria, including consensus meetings and piloting [10]. Limited resources and time within the MRC START programme (of which our work was a part) meant that we did not conduct a Delphi consensus process, and our consensus meetings were each over 1 day only. Nevertheless, we did seek consensus from a wider range of participants.

The item checklist for embedded recruitment trials does not include additional items, similar to CONSORT extensions for reporting specific types of RCTs, which differ little from the CONSORT statement that they were based on, e.g. CONSORT non-inferiority [47], and CONSORT for pragmatic trials [48]. However, we did identify items that need to be considered carefully and reported differently in embedded recruitment trials. These include item 2a—scientific background and explanation of rationale; 4a—eligibility criteria for participants; 4b—settings and locations where the data were collected; 5—interventions; 7a—sample size; 11a–11b—blinding; 12b—additional analyses; 13a—participant flow diagram; 14a—defining recruitment and follow-up periods; 15—baseline data tables; 16—number of participants (denominator) included in each analysis; 19—important harms or unintended effects; 23—trial registration; and 25—funding and other support. Items listed here highlight the need for rigorous design and conduct of embedded methodological trials to ensure unbiased results. We believe these reporting guidelines (particularly the examples) provide a useful tool for those designing embedded recruitment trials.

In developing these guidelines the primary focus was embedded recruitment trials, mainly because the MRC START research programme was initiated to improve recruitment to clinical trials. However, we believe the science underpinning embedded recruitment trials can be extended to other embedded method trials and these guidelines can be applied for those trials.

The primary goal of an embedded recruitment trial may be to facilitate recruitment in the host trial or a specific patient group where recruitment was a particular issue. However, to maximise benefits to the research community, those carrying out embedded recruitment trials should report them in a way which ensures researchers can apply the findings appropriately. We hope the guideline we have presented here will facilitate this and will have a ripple effect on design and conduct of embedded trials as a whole.

## Additional file

Additional file 1:
**Further Examples** **[** 49, 50 **].** (DOC 93 kb)

## Abbreviations

EQUATOR: Enhancing the Quality and Transparency of health Research
HTA: Health Technology Assessment
MRC: Medical Research Council
RCTs: randomised controlled trials
REC: Research Ethics Committee
START: Systematic Techniques for Assisting Recruitment to Trials
