# Supplementary material for: Guidelines for reporting embedded recruitment trials
Source: Trials. 2016 Jan 14;17:27. doi: 10.1186/s13063-015-1126-y (PMC4714476; doi:10.1186/s13063-015-1126-y)
Supplement: Additional file 1: — Further Examples [ 49, 50 ]. (DOC 93 kb) [file 13063_2015_1126_MOESM1_ESM.doc]

# Appendix 1: Further Examples

Table A1: Table showing extension for embedded recruitment trial for each item and additional examples.

| **Item 2a:** **scientific background and explanation of rationale for *embedded recruitment trial including a brief description of the host trial(s) as appropriate***  Example 1  The Systematic Techniques for Assisting Recruitment to Trials (MRC START) research programme is a Medical Research Council (MRC) funded study which seeks to increase the evidence base for recruitment by developing a platform to encourage the rapid and rigorous testing of recruitment interventions. This is done by nesting these recruitment interventions in multiple host trials simultaneously. A fuller description of the MRC START model and study has been provided elsewhere. The two recruitment interventions tested in the first phase of MRC START were optimised patient information material, and access to a multimedia decision aid.  The host trial, Healthlines study was a large primary care research programme consisting of two, multi-centre trials testing the potential clinical and cost-effectiveness of telephone support and computer-based self-management compared to usual care alone. The MRC START optimised patient information materials were nested within the Healthlines Depression and Healthlines CVD RCTs.  Both embedded trials of recruitment interventions were conducted over a four-month period (March 2013 to June 2013) at three primary care practices in the Bristol area (from a total of 43 practices involved in the Healthlines study) [13].  Example 2  The Prostate, Lung, Colorectal and Ovarian (PLCO) Cancer Screening Trial is a 22-year, multisite randomized cancer screening trial funded by the National Cancer Institute. The primary objective of the PLCO Cancer Screening Trial is to determine whether screening for the four PLCO Cancer Screening Trial cancers, which account for 48% of all cancer-related deaths in the United States, decreases mortality from these cancers in adults aged 55-74 years at entrance to the screening. In 1996, the Centers for Disease Control and Prevention and the National Cancer Institute funded an ancillary PLCO Cancer Screening study titled "Recruiting African American Men to the Prostate, Lung, and Colorectal and Ovarian Cancer Screening Trial (the AAMEN Project)".  Despite their higher incidence and mortality of cancer relative to their Caucasian counterparts, African American men are not well represented in cancer screening trials. The AAMEN Project is a randomized trial designed to test the effectiveness of three increasingly intensive recruitment strategies in recruiting African American men to the PLCO Cancer Screening Trial [19]. |
| --- |
| **Item 2b:** **specific objectives or hypotheses *for embedded recruitment trial***  Example 1  The aim of the present study was to assess the impact of on-site initiation monitoring visits on: 1) patients’ recruitment, 2) quantity of data spontaneously reported, 3) quality of data, and 4) patients’ follow-up time [37]. |
| **Item 3a: description of *embedded recruitment trial* design (such as parallel, factorial, *cluster*) including allocation ratio**  Example 1  Member institutions and affiliates of three cooperative oncology groups, Eastern Cooperative Oncology Group (ECOG), North Central Cancer Treatment Group (NCCTG), and Cancer and Leukemia Group B (CALGB), were randomly assigned to either intervention (use of the easy-to-read consent statement) or control (use of the standard consent statement). Because many affiliate institutions share institutional review boards (IRBs), the unit of randomization was the IRB, with the exception of CALGB institutions. CALGB main institutions and their affiliated institutions were assigned together, regardless of shared IRBs. The IRB was chosen as the unit of randomization in non-CALGB institutions to eliminate potential contamination and confusion from using two different consent statements at a single institution [26]. |
| **Item 4a: eligibility criteria for participants *for embedded recruitment trial, including any differences from those for the host trial(s)***  Example 1  Potentially eligible participants were identified from practice lists. For Healthlines Depression, the eligibility criteria were aged at least 18 and a confirmed diagnosis of clinical depression using standardised measures. For Healthlines CVD, participants were aged 40-74, with 20% or greater risk of having a cardiovascular event in the next 10 years (QRISK2 score) and had to have at least one of three modifiable risk factors (systolic blood pressure of 140 or greater; a body mass index (BMI) >=30; or current smoker). Other criteria were access to a telephone, the Internet and having an email address for personal use. There were no additional inclusion or exclusion criteria for the embedded trials [13].  Example 2  The Physical Activity for Total Health study is a randomized controlled clinical trial comparing the effect of a 1-year moderate intensity aerobic and strength-training exercise intervention versus stretching control on endogenous hormone concentrations. We randomized 173 postmenopausal women, ages 50–75 years, who were sedentary (<20 min of exercise 3 times/week) and overweight (body mass index > 25 or percentage of body fat > 33%). Participants resided in the Seattle, Washington area.  At the beginning of the recruitment period, we conducted a pilot study of four mailing strategies and used the results to determine the mailing method for use during the remainder of recruitment. Women targeted for recruitment were identified via lists obtained from the Washington State DMV. Mailings targeted women between the ages of 50 and 75 years, who lived in zip code areas that were less than a 30-min drive from exercise facilities, where the intervention was implemented. Interested women contacted the study by returning a mailed interest survey or calling a designated telephone line, were screened for eligibility by a phone interview, and were asked to attend an information session. Eligible women were scheduled for a screening clinic visit and two treadmill tests to collect baseline data; those who successfully completed the screening process were randomized to an aerobic exercise intervention or stretching control group [23]. |
| **Item 4b: settings and locations where the *embedded recruitment trial was carried out, including a brief description of the host trial(s) as appropriate***  Example 1  This recruitment study was part of a randomized trial at a worksite for staff employees that examined whether the addition of a new goal-setting technique to a standard low-fat dietary intervention resulted in better maintenance of a low-fat diet 6 months later than a standard low-fat dietary intervention alone. Names and worksite addresses for current Stanford University staff employees were generated from a personnel services database and sorted by ethnic group. Hispanic employees were randomly assigned to one of three strategies, described below. All mail was sent via the internal mail at the worksite [25].  Example 2  Three intergroup treatment trials (parent treatment studies: ECOG Protocol E1594: a phase III trial in metastatic non–small-cell lung cancer; CALGB Protocol C9741: a randomized phase III trial of sequential chemotherapy using doxorubicin, paclitaxel, and cyclophosphamide or concurrent doxorubicin and cyclophosphamide followed by paclitaxel at 14- or 21-day intervals in women with node-positive stage II/III breast cancer; and ECOG Protocol E2197: a phase III study of adriamycin/taxotere v adriamycin/cytoxan for the adjuvant treatment of node-positive or high-risk node-negative breast cancer) were selected as parent treatment studies for the purpose of this consent study. Treatment trials were selected based on anticipated high numbers of patients being accrued and the timing of protocol activation being consistent with the activation of this study.  Member institutions and affiliates of three cooperative oncology groups, Eastern Cooperative Oncology Group (ECOG), North Central Cancer Treatment Group (NCCTG), and Cancer and Leukemia Group B (CALGB), were randomly assigned to either intervention (use of the easy-to-read consent statement) or control (use of the standard consent statement) [26]. |
| **Item 5: the interventions for each group *(including control group)* *within the embedded recruitment trial* with sufficient details to allow replication, including how, where and when they were actually administered**  Example 1  The research nurse gave a full explanation of the allocated study design: either the open randomized trial design (participants would be told to which compound they had been allocated, whether vitamin D, calcium, vitamin D and calcium, or no tablets) or the conventional RECORD trial (the randomized blinded, placebo-controlled trial of vitamin D, calcium, vitamin D and calcium, or placebo). The research nurse described the each trial design with information leaflets, which were similar in appearance. Written consent was then collected. The research nurse recorded the exact wording used by people to describe why they did not wish to take part. Tablets and questionnaires were subsequently sent by post from the trial co-ordinating centre. The covering letter and tablet bottles sent to participants in the open trial gave details of their trial allocation. Participants' family doctors also received information about the open trial allocation. At subsequent four monthly intervals participants received further tablets as required by the trial design, and all participants received questionnaires identical in appearance to return by reply paid mail to the trial office [30].  Example 2  We aimed to recruit 5800 participants in two years (242 per month), but in the first 8.5 months we only recruited 1058 participants. The proportion of eligible participants consenting in June 2008 was 35% (1058/3029) whilst the target was 50%.  We identified two points in the recruitment process where participants failed to progress either to randomization or exclusion from the trial. Firstly, we were unable to contact many potential participants who had sent us a mobile phone text message (SMS) to enquire about the trial, because they did not answer their mobile phones. In June 2008 there were 937 potential participants on the ‘outstanding public interest’ list (point A [Figure 3]). Secondly, many eligible participants did not send a text message giving or refusing consent to randomization.  This was a pragmatic trial, so we included all participants on the ‘outstanding public interest list’ for the Txt2stop trial.  Control group: Participants in the control group received the normal trial procedures, which involves research staff calling their mobile number to register them for the trial (no text message).  Intervention group: The intervention was a single text message: ‘Thanks for your interest in Txt2stop, the smoking cessation program. We have tried to contact you but with no luck. You can now register your details at www.txt2stop.org. We will continue to try to speak to you’ [31]. |
| **Item 6a: completely defined pre-specified primary and secondary outcome measures *for the embedded recruitment trial,* including how and when they were assessed**  Example 1  For the embedded recruitment trial of optimised patient information materials in Healthlines Depression, the primary outcome was the proportion of patients randomised. Secondary outcomes were the proportion of patients who accepted the offer of invitation to participate, and the proportion of eligible patients who actively opted out of the trial (i.e. returned a ‘decline’ form).  For the embedded recruitment trial of optimised patient information materials in Healthlines CVD, the primary outcome was the proportion of patients who responded positively to the invitation to participate. This, rather than actual randomisation, was selected as the primary outcome because of a cap on recruitment numbers whereby only the first 25 eligible participants were randomised in each practice. This upper limit was implemented because of practice staff availability to carry out these assessments, and an initial agreement with researchers that 25 patient assessments would be sufficient to reach target recruitment across participating GP practices. The secondary outcome was the proportion of eligible patients who actively opted out (i.e. returned a ‘decline’ form) [13]. |
| **Item 7a**: **how sample size *for embedded recruitment trial* was determined**  Example 1  WIME aimed to recruit 250 GPs. Invitations were sent out until the number of GPs recruited met or exceeded the required sample size of 250 GPs [11].  Example 2  The primary study end point was the percentage of patients in the embedded recruitment trial who declined participation in the host clinical trial that was presented to them. On the basis of the literature, it was assumed that 40% of patients refuse participation in cancer clinical trials, and in order to have an 80% chance of detecting a 20% difference at the 5% significance level (two-sided), a total of 164 patients were required. This number would detect a reduction in refusal rate from 40% to 20%. This sample size also provides approximately 80% power to detect a moderate standardised difference of 0.45 between continuous variables such as knowledge and anxiety scores [22]. |
| **Item 8b: type of randomisation; details of any restriction (such as blocking and block size) *in embedded recruitment trial***  Example 1  Randomization was minimized by age (under 80 years or 80 years and over), sex, time since fracture (previous three months or longer) and type of enrolling fracture (proximal femur, distal forearm, clinical vertebral and other) [30]. |
| **Item 9: mechanism used *in the embedded recruitment trial* to implement the random allocation sequence (such as sequentially numbered containers), describing any steps taken to conceal the sequence until interventions were assigned**  Example 1  After random assignment, institutions randomly assigned to receive educational intervention were notified by the study chair or a member of the trial team [49]. |
| **Item 10: who generated the random allocation sequence(s), who enrolled participants, and who assigned participants to *embedded recruitment* interventions**  Example 1  Standard Healthlines procedure was for lists [of patients] to be screened by GPs and patients with known exclusion criteria removed. A researcher assisted with searches and then randomised patients to receive either the original or optimised patient information materials (using simple randomisation in a 1:1 ratio). Each practice list was randomised separately following the same procedure: each patient was assigned a computer-generated random number using Excel. The list included an ID number for each patient, rather than any identifiable information, thereby reducing the risk of any selection bias on the part of the researcher conducting the randomisation. The list was then sorted by random number, with one half assigned to the optimised patient information materials and the other half to the original [13]. |
| **Item 11a: if done, who was blinded after assignment to *embedded recruitment interventions* (for example, participants, care providers, those assessing outcomes) and how**  Example 1  Investigators were not informed that they would be randomized to be visited or not, for such information might have ruined the very purpose of the study. They were merely told that the trial budget would not allow for regular, extensive on-site monitoring visits such as those typically performed in registration trials of new drugs. Investigators requesting on-site visits were visited regardless of the randomized group their center had been allocated to [37].  Example 2  As clinical sites entered the study (between June 2001 and December 2003) they were informed that most communication would be from the relevant Regional Coordinating Centre but that there may be some communications from the central trial coordinators in Sydney. They were not specifically informed about the conduct of this recruitment study. Regional Coordinating Centres were informed about the study but were blinded with regard to the randomised assignment of each clinical site [29]. |
| **Item 12a: statistical methods used to compare groups for primary and secondary outcomes *of the embedded recruitment trial***  Example 1  We estimated the risk difference and 95% confidence intervals of full registration or consenting to join the trial at 2 weeks using Fisher’s exact test [31]. |
| **Item 12b:** methods for additional analyses, such as subgroup analyses and adjusted analyses *for embedded recruitment trial*  Example 1  A planned secondary analysis was performed to explore whether the impact of the intervention was moderated by gender. This was done by inserting the appropriate interaction term in the logistic regression models [13]. |
| **Item 13a: for each group *in the embedded recruitment trial,* the numbers of participants who were randomly assigned, received intended treatment, and were analysed for the primary outcome**  Example 1  Figure 5 illustrates an embedded trial focused on recruiting participants for host trial [35].  Example 2  Figure 6 illustrates an example where single flowchart was used to show participant flow in both host and embedded trials (It is strongly recommended that number of participants included at each stage must be shown) [33]. |
| **Item 14a: dates defining the periods of recruitment and follow-up for *both embedded recruitment trial and host trial(s)***  Example 1  The ADVANCE (Action in Diabetes and Vascular Disease: Preterax and Diamicron MR Controlled Evaluation) study is a factorial randomised trial addressing questions about optimal blood pressure management and glucose control among high-risk individuals with type 2 diabetes [5,6]. The study originally planned to recruit 10000 participants over 18 months; between July 2001 and March 2003, 11140 participants were recruited from 215 clinical sites in 20 countries in Asia, Australasia, Europe and North America. We used clinical sites involved in this study to evaluate the effects on participant accrual of an indirect recruitment strategy based upon additional communications from the central trial coordinators to the clinical sites.  Our investigation was a single-blind randomised controlled trial conducted within the organisational structure established for the management of clinical sites in ADVANCE. Clinical sites participating in the ADVANCE study were eligible for inclusion if they were from countries able to communicate with the central trial coordinators in English. As clinical sites entered the study (between June 2001 and December 2003) they were informed that most communication would be from the relevant Regional Coordinating Centre but that there may be some communications from the central trial coordinators in Sydney [29]. |
| **Item 14b: why the *embedded recruitment trial* ended or was stopped**  Example 1  The original plan was to include 100 patients, but the study ended early because of declining numbers when several of the randomised clinical trials closed [33]. |
| **Item 15: *if possible* a table showing baseline characteristics of *each arm of the embedded recruitment trial***  Example 1  Table A2 below shows an example where limited baseline data for participants approached was available to researchers [35].  Example 2  Screening was being conducted for two treatment trials of chronic depression at the Payne Whitney Clinic of The New York Hospital-Cornell Medical Center. Patients were recruited by local newspaper advertisements, occasional radio announcements, flyers posted through out the medical center and word of mouth within the psychiatry department. Each respondent to the advertisements received a brief semi-structured telephone interview either by research assistants (RAs) or by a senior investigator (SI). Those who met criteria for an intake assessment based on the phone interview were scheduled for an appointment with a trained clinical rater who administered the complete SCIDP and other assessments [43]. |
| **Item 16: for each group *in the embedded recruitment trial,* number of participants (denominator) included in each analysis and whether the analysis was by original assigned groups**  Example 1  Screening was being conducted for two treatment trials of chronic depression at the Payne Whitney Clinic of The New York Hospital-Cornell Medical Center. Patients were recruited by local newspaper advertisements, occasional radio announcements, flyers posted through out the medical centre and word of mouth within the psychiatry department. Each respondent to the advertisements received a brief semi-structured telephone interview either by research assistants (RAs) or by a senior investigator (SI). Those who met criteria for an intake assessment based on the phone interview were scheduled for an appointment with a trained clinical assessor who administered the complete SCIDP and other assessments [43]. |
| **Item 17a: for each primary and secondary outcome, results for each group *in the embedded recruitment trial,* and the estimated effect size and its precision (such as 95% confidence interval)**  Example 1  A significant difference was observed for the primary end point of attitudes toward clinical trials (P .016; Table A3), reflecting an effect size of 0.46 that favored the MP condition. There were no significant differences by intervention condition for knowledge, self-efficacy, or receptivity to learning more about clinical trials. A statistically significant difference was observed for willingness to participate in a clinical trial (P .011; Table A3), reflecting an effect size of 0.33 that favored the MP condition [50]. |
| **Item 17b: for binary outcomes *in the embedded recruitment trial*, presentation of both absolute and relative effect sizes is recommended**  Example 1  4.5% (11/246) of participants who were sent the letter with £5 were randomized into the Txt2stop trial compared to 0.4% (1/245) of those who were not sent anything. The risk difference is 4.0% (95% confidence intervals 1.4–6.7) [31].  Example 2  The response and recruitment rates for the two methods are shown in table A4 [44]. |
| **Item 18: results of any other analyses performed *for embedded recruitment trial*, including subgroup analyses and adjusted analyses, distinguishing pre-specified from exploratory**  Example 1  There was no evidence that the optimised patient information materials were differentially effective at achieving randomisation among men and women (interaction odds ratio = 0.79, 95% CI 0.27 to 2.28, p = 0.660) [in Healthlines Depression]  There was no evidence that the optimised patient information materials were differentially effective at encouraging a positive response among men and women (Interaction odds ratio = 1.87, 95% CI 0.81 to 4.30, p=0.143) [in Healthlines CVD] [13]. |
| **Item 19: all important harms or unintended effects in each group *for both the embedded recruitment trial and host trial(s)* (for specific guidance see CONSORT for harms [45])**  Example 1  This is the first study to use an experimental design to assess the impact of an open rather than placebo-controlled design on recruitment, compliance and retention. Selection bias was avoided in the comparison of recruitment by randomly allocating eligible people to the designs, and the trial was large enough to identify a plausible difference.  Once recruited, knowledge that a tablet was "active" and knowing what it actually was did not appear to have an effect on compliance amongst those allocated tablets. This should be interpreted cautiously however. The estimates are imprecise and this also assumes that those who did not return their containers were similar to those who did.  Those in the open design were more likely to still be in the trial at one year. In principle, it might be expected that those allocated no treatment in an open design might be most likely to withdraw. In the event, they proved least likely to in this study. The extra withdrawals in the placebo design were due to changing minds, difficulty taking the tablets, or complaining of taking too many tablets. Nevertheless, the differential withdrawal rates in the open group could still introduce more bias; this may offset the advantages of having larger numbers in the analysis [30]. |
| **Item 22: interpretation consistent with results *of the embedded recruitment trial*, balancing benefits and harms, and considering other relevant evidence**  Example 1  Previous work suggests that people choose to participate in trials for a variety of reasons including the extent to which they feel physically threatened by their illness. It is possible that completion of the safety questionnaire raised awareness of the risk of injury and increased recruitment through doing this [44]. |

*Table A2*: Baseline characteristics and recruitment rate for each group [35]

|  | Group 1 | Group 2 | Group 3 | Group 4 |  |
| --- | --- | --- | --- | --- | --- |
|  | n = 140 individuals | n = 140 individuals | n = 140 individuals | n = 140 individuals |  |
|  | (98 households) | (101 households) | (97 households) | (99 households) | Overall |
|  | No telephone, | No telephone, | Telephone, | Telephone, | n = 560 individuals |
|  | no questionnaire | questionnaire | no questionnaire | questionnaire | (395 households) |
| Age |  |  |  |  |  |
| Mean (95% Confidence  Interval) | 73.6 (72.4–74.7) | 72.8 (71.8–73.7) | 73.8 (72.7–74.9) | 73.1 (72.1–74.0) | 73.3 (72.8–73.8) |
| (Standard error) | (0.58) | (0.49) | (0.57) | (0.47) | (0.26) |
| Sex |  |  |  |  |  |
| Number and percentage of  women | 70 (50.0%) | 73 (52.1%) | 76 (54.3%) | 78 (55.7%) | 297 (53.0%) |
| Number and percentage  recruited into physical  activity study | 59 (42.1%) | 47 (33.6%) | 65 (46.4%) | 69 (49.3%) | 240 (42.9%) |

*Table A3*: Adjusted follow-up scores for study outcomes and results for analyses of intervention effects [50]

|  | MP Condition (n = 227) | | PE Condition (n = 235) | |  |
| --- | --- | --- | --- | --- | --- |
| Outcome | Mean | 95% CI | Mean | 95% CI | P |
| Attitudes toward clinical trials | 3.69 | 3.64 to 3.75 | 3.58 | 3.53 to 3.64 | 0.016 |
| Knowledge about clinical trials | 7.91 | 7.66 to 8.15 | 7.62 | 7.37 to 7.78 | 0.353 |
| Self-efficacy for clinical trial decision making | 4.18 | 4.12 to 4.24 | 4.14 | 4.08 to 4.19 | 0.645 |
| Receptivity to clinical trial information | 4.3 | 4.19 to 4.41 | 4.25 | 4.14 to 4.36 | 0.645 |
| Willingness to participate in clinical trial | 3.75 | 3.64 to 3.86 | 3.5 | 3.38 to 3.61 | 0.011 |

*Table A4*: Response and recruitment rates for the invitations with and without the safety questionnaire [44]

| Method | Responded to invitation (%) | RR (95% CI) | Recruited to trial (%) | RR (95% CI) |
| --- | --- | --- | --- | --- |
| Invite with questionnaire (n=1203) | 259 (21.5) | 1.54 (1.29, 1.84) | 217 (18.0) | 1.37 (1.13, 1.65) |
| Invite without questionnaire (n=1190) | 166 (13.9) | χ2=23.53, | 157 (13.2) | χ2=10.65, |
|  |  | 1 df, p<0.001 |  | 1 df, p=0.001 |
